# Genome-Wide Linkage Scan to Identify Loci Associated with Type 2 Diabetes and Blood Lipid Phenotypes in the Sikh Diabetes Study

**DOI:** 10.1371/journal.pone.0021188

**Published:** 2011-06-16

**Authors:** Dharambir K. Sanghera, Latonya F. Been, Sarju Ralhan, Gurpreet S. Wander, Narinder K. Mehra, Jai Rup Singh, Robert E. Ferrell, Mohammed I. Kamboh, Christopher E. Aston

**Affiliations:** 1 Department of Pediatrics, College of Medicine, University of Oklahoma Health Sciences Center, Oklahoma City, Oklahoma, United States of America; 2 Section of Cardiology, Hero DMC Heart Institute, Ludhiana, Punjab, India; 3 Department of Transplant Immunology and Immuno-genetics, All India Institute of Medical Sciences and Research, New Delhi, India; 4 Central University, Bathinda, Punjab, India; 5 Department of Human Genetics, Graduate School of Public Health, University of Pittsburgh, Pittsburgh, Pennsylvania, United States of America; South Texas Veterans Health Care System, United States of America

## Abstract

In this investigation, we have carried out an autosomal genome-wide linkage analysis to map genes associated with type 2 diabetes (T2D) and five quantitative traits of blood lipids including total cholesterol, high-density lipoprotein (HDL) cholesterol, low-density lipoprotein (LDL) cholesterol, very low-density lipoprotein (VLDL) cholesterol, and triglycerides in a unique family-based cohort from the Sikh Diabetes Study (SDS). A total of 870 individuals (526 male/344 female) from 321 families were successfully genotyped using 398 polymorphic microsatellite markers with an average spacing of 9.26 cM on the autosomes. Results of non-parametric multipoint linkage analysis using S_all_ statistics (implemented in Merlin) did not reveal any chromosomal region to be significantly associated with T2D in this Sikh cohort. However, linkage analysis for lipid traits using QTL-ALL analysis revealed promising linkage signals with p≤0.005 for total cholesterol, LDL cholesterol, and HDL cholesterol at chromosomes 5p15, 9q21, 10p11, 10q21, and 22q13. The most significant signal (p = 0.0011) occurred at 10q21.2 for HDL cholesterol. We also observed linkage signals for total cholesterol at 22q13.32 (p = 0.0016) and 5p15.33 (p = 0.0031) and for LDL cholesterol at 10p11.23 (p = 0.0045). Interestingly, some of linkage regions identified in this Sikh population coincide with plausible candidate genes reported in recent genome-wide association and meta-analysis studies for lipid traits. Our study provides the first evidence of linkage for loci associated with quantitative lipid traits at four chromosomal regions in this Asian Indian population from Punjab. More detailed examination of these regions with more informative genotyping, sequencing, and functional studies should lead to rapid detection of novel targets of therapeutic importance.

## Introduction

Type 2 diabetes (T2D) is a major public health problem of 21^st^ century and the fifth leading cause of death worldwide. According to Global Burden of Disease Study predictions, India, China and USA will be the top three leading countries for the prevalence of diabetes [1]. The approximate estimate of 31.7 million people with diabetes in India in 2000 will increase to 79.4 million by year 2030 and the size of the USA population with diabetes, both diagnosed and undiagnosed, will rise from approximately 30 million now to 44 million by the year 2030 [2]. T2D is strongly linked to various metabolic disturbances including obesity, insulin resistance, dyslipidemias, and elevated blood pressure. Linkage and candidate-gene focused studies successfully identified some rare forms of T2D controlled by one or two genes such as the various forms of maturity onset diabetes of young (MODY), mitochondrial diabetes, and neonatal diabetes. However, no single locus was noted to have strong and consistent evidence of linkage with the most common form of T2D in multiple populations [3].

Elevated serum lipid levels are important risk factors for the development of cardiovascular disease (CVD). The genetic basis of several monogenic forms of lipid disorders has been determined, including familial lipoprotein lipase (LPL) deficiency, apoC-II deficiency, defective apoB, familial hypercholesterolemia, and familial triglyceridemia [4]. However, genes associated with common forms of dyslipidemia in the general population remain elusive.

Recent genome-wide association studies (GWAS) performed for many complex traits are revolutionizing the dissection of genetic determinants of several complex traits including T2D and serum lipids. Although these studies are adding to the list of reliably associated common loci controlling T2D and blood lipids and even other complex traits, these loci explain only a small portion of the heritable component associated with these complex diseases. Clearly, additional loci that can explain a large proportion of the variation await discovery.

Asian Indians, one quarter of the global population, have unusually high CVD mortality and very high prevalence of insulin resistance and T2D [5]. The increased susceptibility to early onset of T2D and premature CVD in Asian Indians was confirmed in several earlier studies [6], [7], [8], [9]. Indians tend to develop T2D at a relatively earlier age of 40–45 that is about 10–15 year earlier than European populations [6], [10], [11], [12]. However, the reasons underlying the increased morbidity and mortality associated with T2D and CVD and in people of South Asian ancestry are poorly understood. In this investigation, we have carried out an autosomal genome-wide linkage scan to map the genes associated with T2D and serum lipid levels using our large family-based cohort from the Sikh Diabetes Study (SDS) [13]. This non-smoking, primarily vegetarian, endogamous caste group has high prevalence of diabetes and CVD with young age-of-onset. To our knowledge, this is the first report of genome-wide linkage studies on T2D and quantitative lipid traits in a population from South Asian Continent.

## Methods

### Study Population, Ascertainment Criteria, and Recruitment

This study was carried out on an endogamous community of Khatri Sikhs living in Northern Indian states of Punjab, Haryana, and New Delhi. The Khatri population was chosen because of its relatively higher prevalence of diabetes as compared to other Sikh castes. Khatri Sikhs are more affluent and live in cities and are traders by profession. In general, Sikhs do not smoke for religious and cultural reasons and about 50% of the study participants are life-long vegetarians. A total of 1,115 individuals from 338 families were extensively phenotyped [13]. DNA samples of 870 individuals (526 male/344 female) comprising 685 T2D cases and 185 normal glucose tolerant (NGT) relatives were successfully genotyped and used in this investigation. The T2D cases were 25 years or older and mean age at the time of recruitment (mean ± standard deviation [SD]) was 54.2±11.0 years. Average age of unaffected relative was 46.0±14.7 years with a minimum age of 19 years. Only individuals who reported that all four grandparents were Khatri Sikhs of North Indian origin, who had Khatri surnames, and who spoke the Punjabi language were included. In addition, probands were required to have two or more full siblings with diabetes, or at least one living parent, and more than two siblings available for sampling. Excluded from the sample were half-siblings, adopted individuals, and individuals of South, East and Central Indian origin; individuals with type 1 diabetes (T1D) or a family member with T1D; individuals with rare forms of T2D such as maturity-onset diabetes of young (MODYs), or secondary diabetes (e.g., due to hemochromatosis or pancreatitis). Clinical characteristics of the SDS participants used for this investigation are summarized in **Table 1**. All blood samples were obtained at the baseline visit. All participants provided a written consent following an informed consent procedures approved by Institutional Review Boards (IRBs). All SDS protocols and consent documents were reviewed and approved by the University of Oklahoma Health Sciences Center (OUHSC) (IRB # 13302, approved till August 31, 2011) and the University of Pittsburgh (IRB # 021234) as well as the Human Subject Protection (Ethical) committees at the participating hospitals and institutes in India. The Ethical committees of local institutions in India were Hero DMC Heart Institute, Ludhiana, and Guru Nanak Dev University, Amritsar. Each Institute in India also separately obtained Federal Wide Assurance (FWA) from the Office of Human Research Protection (OHRP) from the US Department of Health and Human Services (DHHS). All the key investigators and key personnel working for SDS obtained online training for Human Participant Protection Education for Research.

**Table 1:** Characteristics of Study Population Stratified by Gender and Disease (Mean ± SD).

		T2D cases	Unaffected Relatives	*p* value**
		685 (412M/273F)	185 (116M/69F)	
Age at recruitment (year)	M*	52.42±11.36	44.71±15.20	<0.0001
	F	55.93±10.88	48.60±13.60	<0.0001
Age at onset (years)	M	45.80±10.65	–	–
	F	48.62±10.40	–	–
Duration of T2D (years)	M	7.82±7.42	–	–
	F	7.33±6.65	–	–
BMI (kg/m^2^)	M	26.90±4.23	26.89±4.53	0.876
	F	28.50±5.20	27.58±4.79	0.140
WAIST (cm)	M	95.50±10.40	93.2±11.55	0.040
	F	92.70±11.00	88.1±10.60	<0.0001
HIP (cm)	M	95.8±8.20	96.0±8.50	0.875
	F	99.3±11.00	97.6±9.70	0.135
WHR^†^	M	0.99±0.07	0.97±0.07	<0.0001
	F	0.94±0.07	0.90±0.07	<0.0001
Fasting Glucose (mg/dl)	M	185.81±70.66	95.30±11.38	<0.0001
	F	193.23±74.58	97.77±9.31	<0.0001
Total Cholesterol (mg/dl)	M	177.18±44.34	174.54±45.86	0.525
	F	187.24±47.42	177.58±38.94	0.049
Triglycerides (mg/dl)	M	197.63±113.50	160.71±85.49	<0.0001
	F	172.66±95.34	155.74±69.72	0.078
HDL-cholesterol (mg/dl)	M	38.11±12.44	39.21±10.01	0.321
	F	41.76±12.59	43.70±11.37	0.187
LDL-cholesterol (mg/dl)	M	99.98±35.68	98.36±32.96	0.619
	F	110.17±39.81	102.89±35.56	0.098
VLDL-cholesterol (mg/dl)	M	39.75±24.05	32.45±18.43	0.001
	F	34.77±19.18	31.13±13.84	0.056

*M - male, F- female; ^†^Waist to hip ratio; **Difference between T2D cases and unaffected relatives.

### SDS Families

A total of 557 families were investigated and 236 families were excluded because they did not meet the eligibility criteria for the study. A total of 321 families containing 870 individuals (526 male/344 females), who were successfully genotyped (call rate >95%), were used for linkage analysis of T2D. These 321 diabetic families comprised 275 affected sibling pairs, 59 affected cousin pairs, 127 affected parent-child pairs, 1 affected grand parent-child pair, and 61 affected avuncular pairs. We collected an average of 6.5 participants per family with family size ranging from 3 to 105 members. The average number of generations per family was 2.5. Of these 321 families, 316 families containing 846 individuals (511 male/335 female) were used in the linkage analysis of blood lipids **(** **Table 2** **)**.

**Table 2:** Description of SDS Pedigrees.

	Phenotyped*			Genotyped and phenotyped for T2D**			Genotyped and phenotyped for lipid levels**		
	Total	Male	Females	Total	Male	Females	Total	Male	Females
Number of families	338	–	–	321	_	_	316	_	_
Family size	6.51(*)	–	–	2.71(**)	_	_	2.68	_	_
Generations (average)	2.46	–	–	2.49	_	_	2.49	_	_
Number of individuals in pedigrees	2,199(*)	1248	951	870(**)	526	344	846(**)	511	335
Founders	979(*)	–	–	85	_	_	82	_	_
Number of affecteds in pedigrees	1,202(*)	710	492	685	412	273			
Number of individuals with blood available	1,115	684	431						
Number of affecteds with blood available	868	530	338						
Number of unaffected with blood available	247	154	93						

*—including deceased; **—excluding deceased.

### Phenotypes

The diagnosis of T2D was confirmed by (a) searching medical records for indications of symptoms of diabetes or measures of blood glucose levels, (b) use of diabetic medication, and (c) measuring fasting glucose levels following the guidelines of American Diabetes Association [14]. A medical record indicating either (1) a fasting plasma glucose level >126 mg/dl after a minimum 12-h fast or (2) a 2-h post glucose level >200 mg/dl [2-h oral glucose tolerance test (OGTT)] on more than one occasion with symptoms of diabetes. In the absence of medical record information, we confirmed self-reported T2D cases by performing a 2-h OGTT. The 2-h OGTTs were performed following the criteria of the World Health Organization (WHO) (75 g oral load of glucose). The NGT diagnosis was based on a fasting glycemia <108 mg/dl or a 2-h glucose <140 mg/dl. The average age at diagnosis was 47 years and duration of diabetes was about 7.5 years. Since T2D remains asymptomatic for several years, an average Asian Indian patient with new onset of diabetes might actually had diabetes 4–7 years before diagnosis [15]. This is in sharp contrast to the mean age at onset of 60 years or above in developed countries [10], [11], [16], [17].

Body mass index (BMI) was calculated as [weight (kg)/height (meter)^2^], and waist-to-hip ratio (WHR) was calculated as the ratio of abdomen or waist circumference to hip circumference. Despite having comparable BMI (27.5±4.0 T2D cases vs. 27.3±4.7 controls), patients had a pronounced abdominal adiposity as reflected by their significantly higher WHR (0.97±0.07 vs. 0.94±0.07; p<0.0001) than controls. Interestingly, WHR in Khatri Sikh men (BMI 26–27 kg/m^2^) was higher than obese Mexican American men (BMI>32 kg/m^2^); Sikhs (0.97±0.05) vs. Mexican Americans (0.95±0.06) [18]. Perhaps central obesity is the underlying cause of high risk to insulin resistance and high prevalence of T2D and CVD in Indians.

Education (highest level completed) was scored 1–4 where 1 =  primary or none, 2 = high school, 3 = bachelor degree, and 4 =  post graduate degree. Job-grade was scored 1–3 based on education and economic status where 1 =  high income, 2 =  middle-income, 3 =  lower-middle and lowest income class; category 1 was used as a reference group. Smoking information was collected on past smoking, current smoking status, length of time, number of cigarettes smoked/day. Alcohol consumption was scored 0–4 where 0 =  no alcohol, 1 =  50 to 100 ml/day, 2 =  100 to 400 ml/day, 3 =  400 to 1000 ml (1L)/day 4 = >1 L/day. Physical activity was scored 1–3 based on level of activity performed where 1 =  very active, 2 =  moderately active, 3 =  quite inactive. About 83% of T2D patients were taking oral hypoglycemic agents. Some were maintaining glycemic control by diet and exercise. The individuals on lipid-lowering medications were not included in the analysis. Further recruitment details are available elsewhere [13].

### Metabolic Estimations

Serum lipids [total cholesterol, high-density lipoprotein (HDL) cholesterol, low-density lipoprotein (LDL) cholesterol, very low-density lipoprotein (VLDL) cholesterol, and triglycerides] were quantified using standard enzymatic methods (Roche, Basel, Switzerland). Fasting serum insulin was measured by radio-immuno assay (Diagnostic Products, Cypress, USA). All quantitative parameters were determined by following manufacturer's instructions using a Hitachi 902 auto-analyzer (Roche, Basel, Switzerland).

### Marker Genotyping

DNA was extracted from buffy coats using QiaAmp blood kits (Qiagen, Chatworth, USA) or by the salting out procedure [19]. 870 samples were successfully genotyped for 398 polymorphic microsatellite markers with an average spacing of 9.26 cM on the autosomes by the National Heart Lung and Blood Institute's (NHLBI) Mammalian Genotyping Service (http://www.marshmed.org/genetics). A total of 870 (526 male, 344 female) samples were used in linkage analysis of T2D and 846 (511 male, 335 female) samples were used in linkage analysis of lipid levels after excluding those with call rate <95%, relationship errors, gender errors, and those with missing phenotypes.

### Error Checking and Data Handling

A variety of statistical software was used to complete this study. To set up the files for analysis, we extensively used the statistical software R (version 2.0.1). Data cleaning was performed following several steps. To check for inconsistencies in the self-reported family structures, we carried out relationship testing using PREST [20] and RELPAIR [21], [22]. PEDCHECK [23] was used to detect Mendelian inconsistencies in genotype combinations within a family. PEDSTATS (version 0.6.9) [24] was used to obtain counts of individuals included in the analysis.

### Phenotype Normalization and Adjustment for Covariates

To adjust for the confounding effects of environmental influence on the lipid traits, we included information on age, age^2^ sex, BMI, dietary and lifestyle factors (smoking, alcohol consumption, and physical activity), socio-economic status (education and job-grade) as covariates. To select significant covariate, both stepwise regression and backward elimination were used in genetic models. Significant covariates considered for selection in the model were age, age^2^, sex, job grade, level of alcohol consumption. Additionally, analysis was performed including and excluding BMI in the model despite its elimination in stepwise regression. Univariate analysis was performed to obtain summary statistics for each trait (online supplementary Table S2). A classical multiple linear regression model: 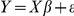, was used where 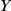 is the response and 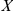 is the design matrix and 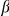 is the vector of regression coefficients. To reduce collinearity between age and age^2^, these variables were mean centered. Since most of the traits were right skewed, they were transformed using the Box-Cox transformation method (Figure S1). Box-Cox method provides optimal value of the transformation parameters and increases the applicability and usefulness of statistical techniques based on the normality assumption and can significantly improve the linear fit of Y against X . After transformation we checked for the outliers. Regression parameters were estimated after exclusion of outliers (points outside of mean ±3 SD), and residuals were computed for all participants. After building the model, the data were rechecked for further outliers using the jackknife method. Influential observations also were taken care of. High Leverage point and Cook's distance for each observation were also measured. To test the significance of the parameters, a significance level of 0.05 was used throughout the analyses. To sample a set of putatively unrelated individuals for the regression analysis, we took all phenotyped founders. If a family had no phenotyped founders, then we sampled one phenotyped non-founder from that family. Although BMI was not a significant covariate in step-wise selection, the entire QTL-ALL analyses were performed both including and excluding BMI as a covariate in the model.

### Genome-wide Linkage Analysis for T2D

We used the S_all_ statistic [25] as implemented in Merlin [26] to perform linkage analysis for the trait T2D. This non-parametric method has excellent power and is robust across a wide variety of disease models [27], [28]. Using FastSLINK [29], [30], [31], we simulated genetic data, for a 2-allele marker for an allele frequency of 0.01 and a penetrance vector of (0.054, 0.50, 0.70), which implies that the relative risk to siblings, λ_s_, is a relatively low 1.49. Then a S_all_ LOD score was computed using Merlin. From this simulated data we have 98% power to detect a LOD > = 2; and 90% to detect a LOD > = 3 in this cohort. Notably, these power estimates are conservative as we excluded the biggest two families which were too complex to run through Merlin.

### QTL-ALL Analysis for Mapping Lipid Traits

In this study, families containing individuals affected by T2D are preferentially over-sampled, so this sample is non-randomly ascertained with respect to T2D. Therefore, to the extent that lipid traits are correlated with T2D status, the sample is also non-randomly ascertained with respect to the lipid traits. Thus, it would not be appropriate to use the usual variance-component based linkage analysis methods on these data. Instead we used score-based linkage statistics as implemented in the QTL-ALL program for this data set [32]. We decided to use the statistic SCORE.MAX, which is recommended in most circumstances, and which has been shown to work well even on non-randomly ascertained data. The current version of QTL-ALL can handle only nuclear family pedigrees. So the Mega2 program was used to convert the multi-generation families to single generation nuclear families [33].

## Results

### Family Structure Error, Gender Error and Genotype Error Checking

Family structure data and X-linked genotypes at 27 markers were combined to detect possible gender errors by looking for males who are more heterozygous than expected and females who are more homozygous than expected. Five males were heterozygous at more than two markers; 16 women were more than 80% homozygous. All suspect participants were rechecked to ensure there was no misreporting of gender. We used RELPAIR and PREST to check the accuracy of self-reported family relationships. Misclassification of relationship for half-siblings as full-sibling, and unrelated as cousins, were detected and resolved. Participants with unresolved relationship errors were removed from families before analysis. We also used PEDCHECK to check Mendelian inconsistencies at each marker and erroneous data were omitted from further analysis. **Table 1** shows the clinical and physical characteristics of the SDS participants used in the analysis.

### Linkage Analysis for T2D

As shown in online **Figure S1**, non-parametric multipoint linkage analysis did not show any chromosomal region to be significantly associated with T2D in this Sikh cohort. Adjusting for age, BMI, and gender did not alter linkage signal significantly and consequently were not included as covariates in the results presented. We found little evidence of linkage with T2D with maximum LOD of 1.24 reached on chromosome 2p24 near microsatellite markers SRAP and X130YG9P. No other region revealed any signal (LOD >1.00) associated with T2D in these families.

### Influence of Environmental Factors on Lipid Traits

Univariate analysis of the lipid traits showed some individuals with very high or very low outlier values, which were removed from the analysis. As needed a Box-Cox transformation was used to make the error distribution of the data more normal **(online Figure S2).** Regression models were then fitted for the transformed traits. In the variable selection step, in most cases forward stepwise-regression and backward elimination agreed with each other. **Table 3** shows the final models selected after detecting the significant covariates for each lipid trait analyzed. Total serum cholesterol levels were influenced by economic status. The correlation between VLDL cholesterol and triglycerides was very high (0.98) and level of alcohol consumption was a significant factor for influencing both serum triglycerides and VLDL-cholesterol levels. Gender was a significant covariate for serum HDL cholesterol, and age, age^2^, and socio-economic status (job grade) were significant predictors of serum LDL cholesterol levels **(** **Table 3** **)**. All the estimated coefficients are presented in online **Table S1**. High leverage points and Cook's distance were calculated to detect influential observations and poorly fitted observations. After removing the maximum Cook's distance points, there was no significance change in the model. Calculated jackknife statistics was also within the acceptance region. Residuals of each trait were calculated and these residuals were used for the final QTL-ALL analysis.

**Table 3:** Final model variables in the five lipid traits.

Covariate Trait	Job grade	Alcohol consumption	Sex	Age	Age^2^
Total Cholesterol	*				
Triglycerides		*			
HDL Cholesterol			*		
LDL Cholesterol	*			*	*
VLDL Cholesterol		*			

*represents significant covariate used for each lipid trait.

### QTL-ALL Analysis for Mapping Lipid Traits

**Figure 1:**
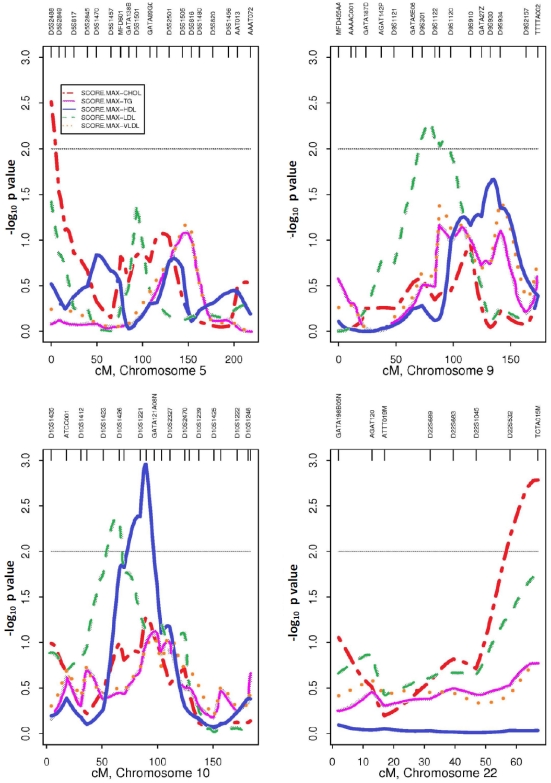
Genome-wide linkage scan to detect susceptibility loci for five blood lipid phenotypes using QTL-ALL analysis using 316 pedigrees. Linkage plots show significant signals at four chromosomal regions with allele sharing LOD (−log10 p value) on Y axis and chromosome distance (cM) on X axis. Significant linkage includes chromosome 5 near marker D5S2488 (p = 0.0031) for total cholesterol; chromosome 9 near marker D9S1122 (p = 0.0039) for LDL cholesterol; chromosome 10q21.2 near D10S1225 (p = 0.0011) for HDL cholesterol; and chromosome 22 near marker TCTA015M (p = 0.0016) for total cholesterol.

QTL-ALL analysis, using the Score.Max statistics, was performed for the five quantitative traits. An overview of the linkage results for the significant signals associated with serum lipid associated traits is given in **Figure 1** and **Table 4**. Several QTLs with p≤0.005 were detected on chromosomes 5p, 9q, 10q, 10p, and 22q. The strongest linkage signal (p = 0.0011) was detected on chromosome 10q21.2 near D10S1225 for serum HDL cholesterol. Suggestive evidence of linkage for total cholesterol was observed on chromosome 5 near marker D5S2488 (p = 0.0031), and on chromosome 22 near marker TCTA015M (p = 0.0016). Two signals, one near marker D9S1122 (p = 0.0039) on chromosome 9 and other near D10S1426 (p = 0.0045) on chromosome 10, were detected for LDL cholesterol. A peak for HDL (p = 0.031) was seen near marker D9S934 on chromosome 9. No significant signal for serum triglycerides was observed **(online Figure S3)**. Because obesity is a major risk factor for CVD and T2D risk, and affects lipid levels, we also tested linkage signals including and excluding BMI. Our results did not change after including BMI in the model.

**Table 4:** Susceptibility regions for serum lipid levels with Score.Max p values of ≤0.005.

Trait	Chromosome	Cytogenetic position	Physical Position*	Closest Marker	Genetic Position (cM)	Score.Maxp Value
Total Cholesterol	5	5p15.33	180390	D5S2488	0.00	0.0031
LDL Cholesterol	9	9q21.13	78878414	D9S1122	75.88	0.0039
LDL Cholesterol	10	10p11.23	30535660	D10S1426	65.61	0.0045
HDL Cholesterol	10	10q21.1	57199892	D10S1221	84.44	0.0041
HDL Cholesterol	10	10q21.2	64425005	D10S1225	89.69	0.0011
Total Cholesterol	22	22q13.32	47925896	TCTA015M	66.96	0.0016

*NCBI Build 36.1 positions from the UCSC browser.

## Discussion

Our study represents the first large scale genome-wide effort to identify chromosomal regions with putative loci affecting T2D and lipid traits in a unique community of Asian Sikhs from Northern India. This diabetic cohort from a genetically homogenous subgroup was collected with the initial goal of identifying T2D predisposing genes. However, the results of our non-parametric linkage scan did not identify any chromosomal region to be significantly linked to T2D **(online Figure S1)**. Note that the non-parametric method for linkage (used in our study) only considers allele sharing between affected individuals, therefore, the ambiguous phenotype of unaffected members is unlikely to have led to the failure to detect linkage in this large sample. These results reaffirm the highly complex nature of T2D phenotype. Essentially, our study failed to identify genes associated with T2D even when a homogenous population was used to control genetic heterogeneity associated with T2D phenotype and a sample collected from one geographic location was used to reduce environmental heterogeneity. These finding suggest that the genes responsible for T2D in Sikhs have small effects, as seen in other ethnic groups, and are difficult to detect using linkage analysis. It can be argued that in comparison to random-mating population, higher identity by descent (IBD) sharing in this inbred population might have reduced the power of detecting significant linkage. In this scenario, one would expect to see increased average IBD leading to false positive indications of linkage. On the contrary, we found the opposite with no substantial increase in IBD among affected individuals and thus no linkage. At the same time, we believe that our linkage data may still contain considerably useful information that could enable the discrimination of causal variant from a near-by variant that is merely in linkage disequilibrium (LD) [34]. Interestingly, our case-control association studies have confirmed some Caucasian GWAS loci (*TCF7L2, PPARG, KCNJ11, FTO* and *KCNQ1*) associated with T2D in this population [35], [36], [37], [38]. Therefore, further fine mapping especially in the elevated regions using high-density SNP panel and whole genome sequencing may identify rare and functional variants with large effects contributing to T2D. These Investigations also may answer the questions of ‘missing heritability' which is expected to lie in the ‘rare' variants and which the GWA studies are unable to explain [39].

The other aim of this investigation was to identify genomic regions affecting lipid-related phenotypes in this cohort. We performed QTL-ALL analysis on this non-randomly ascertained dataset, which revealed several suggestive linkage signals associated with serum lipid levels **(** **Table 4** **)**. Classical multiple linear regression models were used to adjust for environmental effects on the serum lipid traits. In view of strong environmental component associated with T2D and lipid metabolism, we have carefully analyzed the environmental factors, particularly the unique life style factors such as diet, physical activity, obesity, job status, socio-economic status, gender, and medication that could potentially influence these traits. As explained in the Results section, the significant covariates with potential to modify linkage effect were identified and included in the analysis model. The strongest evidence of linkage (p = 0.0011) for HDL cholesterol was detected on chromosome 10q21.1–21.2. Suggestive evidence of linkage to ApoA-I was observed on chromosome 10q21.1 in the Quebec Family Study (QFS) [40]. The same region containing protocadherin 15 (*PCDH15)* gene (10q21.1) has been associated with multiple lipid traits in Finnish and Dutch multigenerational dyslipidemic families [41]. Another strong GWAS candidate gene linked with metabolic traits is solute carrier family 16, member 9 (*SCL16A9)* that also maps to chromosome 10q21.2 [42]. It is a proton-linked monocarboxylate transporter and catalyzes the rapid transport of many monocarboxylates across the plasma membrane. Chromosome 10 also carried a signal for LDL cholesterol at 10p11.23 (p = 0.0045) in our study. The closest candidate gene at this region is *KIAA1462* (10p11.23) that encodes a yet uncharacterized protein. However, a recently published GWAS showed an unambiguous evidence for association of rs3739998 (p = 7.2×10^−8^) within this gene with CVD and myocardial infarction in German MI Family cohort (GerMIFS) III (KORA) [43]. Interestingly, some common variants in *PCDH15* and *SCL16A9* and *KIAA1462* genes are also associated with multiple lipid traits including HDL cholesterol, LDL cholesterol, and triglycerides ( p values of 0.004 to 0.0001) in our provisional results of lipid GWAS being performed on the population originated from the same Asian Indian community (unpublished results).

A linkage peak for total serum cholesterol (p = 0.0031) was detected near marker D5S2488 at the proximal region of chromosome 5p15.33. This region was previously linked to LDL cholesterol in the NHLBI Family Heart Study [44] and HDL cholesterol in the Hypertension Genetic Epidemiology Network Blood Pressure Study [45]. Additionally, meta-analysis of linkage scans from four studies revealed a modest signal for LDL cholesterol (LOD 1.6) on chromosome 5p15.33 [46]. The suggestive linkage for LDL cholesterol at 9q21.13 near marker D9S1122 in our cohort was also associated with the triglyceride phenotype in a linkage study performed in families with myocardial infarction [47].

The linkage signal at chromosome 22q13.32 near marker TCTA015M (p = 0.0016), detected for total cholesterol was linked with familial hypercholesterolemia in a Utah study [48]. The chromosomal region 22q11–13 was also reported to effect HDL cholesterol in the Old Order Amish [49]. Notably, the strongest candidate gene in this region is *PPARα*, which is a ligand-activated nuclear transcription factor and controls extracellular and intracellular lipid metabolism, and also inhibits progression of atherosclerotic lesions [50]. Lipid-lowering drugs of fiberate class are synthetic ligands of pparα [51]. Variants in this gene were reported to be associated with T2D and CVD [52]. Another gene *CELSR1* (located at 22q11–13) is associated with ischemic stroke in recent Japanese GWAS [53]. Furthermore, a single nucleotide polymorphism (SNP) near *CELSR2* on chromosome 1p13 (homologous to *CELSR1*) is associated with LDL cholesterol and myocardial infarction in a meta-analysis study by Myocardial Infraction Genetics Consortium [54].

Our study does not represent a common replication attempt to identify lipid loci in an independent population. Rather, this investigation has been carefully carried out in this unique family-based cohort using a conservative statistical approach applying score-based statistics to map quantitative lipid traits in a non-randomly ascertained dataset. Exceeding our expectations, this study has identified linkage regions, primarily HDL cholesterol (10q21.1–21.2) and total cholesterol (22q13.32) that were previously reported for lipid traits or CVD. The most interesting part of this study is that some of these linkage signals also harbor important candidate loci (e.g., *KIAA1462, PCDH15, PPARα, SLC16A9,* and *CELSR1*) implicated with lipid traits in recent GWAS and meta-analysis studies and also some of these regions overlap with prior linkage studies [55], [56], [57]. Therefore, our findings suggest that these regions might contain some novel genes for blood lipids rather than chance findings, and perhaps some of the loci may have larger effects in this Khatri Sikh cohort. Notably, the presence of HDL cholesterol signal on chromosome 10q21.2 is particularly important in view of low HDL cholesterol-associated CVD risk in Asian Indian men, in general, and may strongly relate to gene-environmental interaction which is enhanced by rapidly emerging western lifestyle [58], [59]. Further fine mapping with more efficacious strategy using SNP-based arrays (which would also help determine LD over small intervals), sequencing, and functional studies should allow rapid detection of novel target genes of therapeutic importance under these candidate regions.

## Conclusions

Unlike previous studies, our genome-wide linkage scan could not identify any significant chromosomal region associated with T2D in this unique family cohort of Punjabi Sikhs with increased risk to developing T2D and cardiovascular illnesses. Our study, however, for the first time provides an evidence of linkage for loci controlling quantitative lipid traits at four chromosomal regions in this Asian Indian population. The strongest linkage signal was seen for HDL cholesterol on chromosome 10q21.2. Our data also revealed linkage signals for total cholesterol on chromosome 5p15.33 and 22q13.32, and for LDL cholesterol on 10p11.23 and 9q21.13. Some of these regions have been linked to lipid-related traits in recent GWA studies and contain other plausible candidate genes. The strongest peak for HDL cholesterol (p = 0.0011 at 10q21.2) suggests that this region may contain novel gene(s) influencing serum HDL cholesterol levels and other lipid traits. Further denser and more informative genotyping in each of these regions would be important to discover functional loci influencing blood lipids.

## Supporting Information

Figure S1 Genome-wide non-parametric linkage scans for type 2 diabetes using 321 diabetic pedigrees and 398 microsatellite markers (9.26 cM). Individual plot shows linkage signals (Kong and Cox LOD score) on Y axis and microsatellite markers on X axis. None of the chromosome regions revealed any signal associated with T2D in these pedigrees.
(TIF)

Figure S2 Plot of Box-Cox coefficient lambda and the distribution of five quantitative traits including total cholesterol, triglycerides, HDL cholesterol, LDL cholesterol, and VLDL cholesterol before and after transformation.
(TIF)

Figure S3 Genome-wide autosomal linkage scan for five blood lipid phenotypes. Individual plot shows allele sharing LOD (−log10 p value) on Y axis and chromosome distance (cM) on X axis.
(TIF)

Table S1 Linear regression model for quantitative traits.
(DOC)
