# Supplementary material for: Genome-Wide Linkage Scan to Identify Loci Associated with Type 2 Diabetes and Blood Lipid Phenotypes in the Sikh Diabetes Study
Source: PLoS One. 2011 Jun 16;6(6):e21188. doi: 10.1371/journal.pone.0021188 (PMC3116872; doi:10.1371/journal.pone.0021188)
Supplement: Table S1 — Linear regression model for quantitative traits. (DOC) [file pone.0021188.s004.doc]

| **Table S1: Linear regression model for quantitative traits** | | | | | | |
| --- | --- | --- | --- | --- | --- | --- |
| Total Cholesterol | | | | | | |
|  | Estimate | Std. Error | t value | | Pr(>|t|) | |
| (Intercept) | 17.8277 | 0.2145 | 83.11 | | 0.0000 | |
| *factor(JOBGRADE)2 | -0.5678 | 0.2464 | - 2.30 | | 0.0218 | |
| factor(JOBGRADE)3 | -0.8109 | 0.3749 | - 2.16 | | 0.0312 | |
| Triglycerides | | | | | | |
|  | Estimate | Std. Error | t value | | | Pr(>|t|) |
| (Intercept) | 17.9358 | 0.0581 | 308.90 | | | 0.0000 |
| *factor(ALCOHOL)1 | 0.5678 | 0.1705 | 2.44 | | | 0.0153 |
| factor(ALCOHOL)2 | 0.1365 | 0.1901 | 0.72 | | | 0.4733 |
| factor(ALCOHOL)3 | 0.3243 | 0.5659 | 0.57 | | | 0.5670 |
| factor(ALCOHOL)4 | 0.8541 | 0.5659 | 1.51 | | | 0.1321 |
| HDL Cholesterol | | | | | | |
|  | Estimate | Std. Error | t value | | Pr(>|t|) | |
| (Intercept) | 7.8670 | 0.1042 | 75.48 | | 0.0000 | |
| *factor(SEX)2 | 0.7651 | 0.1541 | 4.97 | | 0.0000 | |
| LDL Cholesterol | | | | | | |
|  | Estimate | Std. Error | t value | | Pr(>|t|) | |
| (Intercept) | 39.0725 | 1.0466 | 37.33 | | 0.0000 | |
| (AGE – mean age) | 0.0312 | 0.0402 | 0.78 | | 0.4377 | |
| (AGE – mean age)^2^ | 0.0075 | 0.0031 | 2.43 | | 0.0155 | |
| *factor(JOBGRADE)2 | 3.2863 | 1.1536 | 2.85 | | 0.0047 | |
| factor(JOBGRADE)3 | 5.7684 | 1.7451 | 3.31 | | 0.0010 | |
| VLDL Cholesterol | | | | | | |
|  | Estimate | Std. Error | | t value | Pr(>|t|) | |
| (Intercept) | 9.8853 | 0.0578 | | 170.97 | 0.0000 | |
| *factor(ALCOHOL)1 | 0.4995 | 0.1721 | | 2.90 | 0.0039 | |
| factor(ALCOHOL)2 | 0.1393 | 0.1897 | | 0.73 | 0.4632 | |
| factor(ALCOHOL)3 | 0.3253 | 0.5645 | | 0.58 | 0.5648 | |
| factor(ALCOHOL)4 | 0.8434 | 0.5645 | | 1.49 | 0.1361 | |

* JOBGRADE (factor 1), ALCOHOL (factor 0), SEX (factor 1) were considered as reference.
